# Additional Cases Illustrating the Modus Operandi of Colchicum in the Cure of Gout

**Published:** 1814-12

**Authors:** John Want

**Affiliations:** Surgeon to the Northern Dispensary. North Crescent


*For the Medical and Physical Journal.*

### *Additional Cases illustrating the Modus Operandi of Colchicum in the Cure of Gout;* by Mr. WANT.

THE following cases are an important addition to those already published, in illustration of the fact that the purgative quality of the Colchicum is not essential to its curative efficiency. It will be remarked, that in the first of them the paroxysm of gout was almost immediately removed by it, *and that the strongest purgatives had been previously administered without effect.*

I am thankful to Dr. Sutton for having exerted my attention to the utility of recording these cases, which, in all probability, I should not have thought worthy of publication, had not doubts been started respecting the modus operandi of the remedy. The accuracy of the statements may be relied on, if the patients themselves are worthy of belief, as they are drawn up nearly in their own words.

On Saturday, the 19th of this month, about two o'clock, I was sent for to Mr. Whitehead, printer, in Whitefriars, who was then labouring under a severe attack of gout in his right hand and elbow, both of which were swollen, inflamed, and excruciatingly painful. It was then beginning in the left finger. I had not quitted him an hour, before it attacked him with great violence in the left hand, both knees, and left foot, which seemed to him to be dislocated. The symptoms rapidly increased in severity until five o'clock, when he could not stand or put his foot to the ground; and, although a robust man, fainted from the violence of the pain. At half past six or seven, he took two drams of the tincture: he then lay on a sofa; and in the course of an hour was so far relieved from pain, that, although he could not previously bear the hand last affected in a dependant posture, he was now enabled to reach his crutch, to support it under his arm, and walk with assistance to bed at eleven. At two in the morning, the pain was scarcely perceptible, if any; but he took a second dose of the medicine by mistake. At six he awoke, got out of bed with perfect facility, and had one very trifling motion; about nine had a

 second; second; at half past ten a third; and at twelve a fourth: the purgative operation was very mild, much more so than that of the Epsom salts, nor were the evacuations so watery, or attended with so much griping. He has been much in the habit of taking opium, which, in the dose of two grains, always affects the head the day after its use, but no inconvenience whatever has arisen from the Colchicum. He has not been sick, and his appetite remains unimpaired. The pain subsided long before the evacuations took place.

The history of the case, previous to my attendance, is as follows. He had been many years subject to Gout. The present attack commenced on Monday the 14th, with pain, swelling, and redness in the thumb, and a feeling as if it were sprained. In the morning of Tuesday he took Epsom salts; in the evening was so ill that he took a purgative draught, with two grains of opium. The medicine operated violently, producing very frequent, copious, and watery, evacuations. Wednesday he was rather easier, took another dose of salts, and in the evening more opium. He steeped his hand in hot water, and smeared it with oil, which seemed to relieve it. Thursday he was rather better, and repeated his salts. Friday afternoon the pain was very violent in his hand, it then flew to the elbow; took two grains and a half of opium; pain increased until I saw him on Saturday, at two o'clock, as above mentioned.

My attendance was desired upon Sophia Sargett, a girl fourteen years of age, residing at No. 7, Abbey-place, Coram-street, on Tuesday, Nov. 23. Five weeks previous to this time she was seized with a diarrhœa, which in three days abated, but violent pains in the legs, feet, and knees, supervened, attended with inflammation and swelling; she was unable to stand or lift the slightest weight. In this state she continued a week without any change, excepting that the pains somewhat abated in violence; the amendment was soon changed for a more severe attack, but in the course of three or four days she was again better. It was always observed, that, as the swelling increased, the acuteness of the pains subsided; on the contrary, when it disappeared the pains returned, and the disease continued alternately better and worse during the whole period of her illness. On Tuesday last the pains were very violent in the legs and ancles; in two or three days the tumefaction appeared, with trifling abatement of pain, as usual; on Friday night the pains recurred in the arm and shoulders; Saturday the hand was affected; Sunday the hip was attacked with great pain and swelling, in which situation she continued until Tuesday morning, when I was desired to see her. Not being able able to visit her at home, I prescribed the ordinary dose of the remedy, and promised to see her on the following day, certainly not expecting so severe an affection of the hip could be removed in a period so short; on the following morning the girl was brought to me quite well, being able to walk to my house. The *medicine produced no effect on the bowels, but twice occasioned slight sickness.*

Mr. Flynn, carver and gilder, Stacey-street, was affected with gout in one of the feet. The attack was slight, but as the symptoms were on the increase, he applied for my advice. I prescribed two drams of the tincture, which was taken at ten o'clock in the morning: before five in the evening the pain was entirely removed; at nine, *no evacuation or disturbance had been created by the medicine.* I have since found, that about twelve, the patient having taken a hearty supper, sickness arose, and the bowels were slightly relaxed; but this was at least seven hours *after* the disease was removed.

JOHN WANT,
*Surgeon to the Northern Dispensary.*

*North Crescent, Nov.* 24, 1814.

