# Errata

**Published:** 1815-01

**Authors:** 


					ERRATA.-
-In our last, p. 491, I. 17, for exerted, read excited

				

